# Supplementary material for: NOTCH activity differentially affects alternative cell fate acquisition and maintenance
Source: eLife. 2018 Mar 26;7:e33318. doi: 10.7554/eLife.33318 (PMC5889214; doi:10.7554/eLife.33318)
Supplement: Supplementary file 1. [file elife-33318-supp1.docx]

| Gene Name | Fd primer | Rv Primer | Amplicon Size |
| --- | --- | --- | --- |
| Hes1 | TGCCAGCTGATATAATGGAGAA | CCATGATAGGCTTTGATGACTTT | 126 |
| Hes5 | GATGCTCAGTCCCAAGGAGA | AGCTTCAGCTGCTCTATGCTG | 96 |
| Hey1 | CATGAAGAGAGCTCACCCAGA | CGCCGAACTCAAGTTTCC | 106 |
| Hey2 | GTGGGGAGCGAGAACAATTA | GTTGTCGTTGAATTGGACCT | 68 |
| HeyL | TAGTCCCAATCCCACCATGT | GAACCAGACACATTGTCATCAGA | 73 |
| Prop1 | ACGAAGGGTCATGCCTGT | CTATAAGCCTCAGAGCTCCTGTCT | 95 |
| Pou1f1 | ACCACAGTGCCGCTGAGT | GTAATGAAGTCCTGTCGCTGTG | 77 |
| GH | GCTTGGCAATGGCTACAGA | GGAAAAGCACTAGCCTCCTG | 91 |
| Prl | TCACCATGACCATGAACAGC | ATCAGCAACAGGAGGAGTGTC | 64 |
| Tshb | AAGAGCTGGGGTTGTTCAAA | ACAAGCAAGAGCAAAAAGCAC | 67 |
| Lhb | CGGCTACTGTCCTAGCATGG | GGAAAGGAGACTATGGGGTCTAC | 144 |
| Pomc | CAGTGCCAGGACCTCACC | CAGCGAGAGGTCGAGTTTG | 72 |
| Pax7 | GGCACAGAGGACCAAGCTC | GCACGCCGGTTACTGAAC | 60 |
| Tbx19 | TGAAATGATCGTGACCAAGAACGG | TTCACCATTGACGTACTTCCAGCG | 144 |
| Neurod1 | ATGACCAAATCATACAGCGAGAG | TCTGCCTCGTGTTCCTCGT | 118 |
| Acsl1 | TCTCCTGGGAATGGACTTTG | GGTTGGCTGTCTGGTTTGTT | 141 |
| Sox2 | AACGCCTTCATGGTATGGTC | GATCTCCGAGTTGTGCATCTT | 78 |
| Sox9 | AGCTCACCAGACCCTGAGAA | CTCCAGCAATCGTTACCTTC | 205 |

SUPPLEMENTARY TABLE 1

­­
